# The second digital divide mediates the association between parental rearing style and depression, anxiety and their comorbidity among college students: a latent profile and mediation analysis

**DOI:** 10.3389/fpsyt.2026.1838943

**Published:** 2026-08-19

**Authors:** Qianqian Xu, Zhikai Yu, Zhiyong Xu, Yi Li, Aihua Tan, Jie Yu

**Affiliations:** 1 Department of Radiology, Zhongnan Hospital of Wuhan University, Wuhan University, Wuhan, Hubei, China; 2 School of Medicine and Health Management, Tongji Medical College, Huazhong University of Science and Technology, Wuhan, Hubei, China; 3 School of Chinese Language and Literature, Hubei University, Wuhan, Hubei, China; 4 Digestive Endoscopy Center, The People’s Hospital of Ying Shan, Huanggang, Hubei, China; 5 Emergency Department, Huanggang Central Hospital, Huanggang, Hubei, China; 6 Central Laboratory, HuangGang Hospital of Traditional Chinese Medicine, Huanggang, Hubei, China

**Keywords:** anxiety, comorbidity, depression, latent profile analysis, parental rearing style, the second digital divide

## Abstract

**Background:**

Depression and anxiety symptoms among college students have become major public health concerns worldwide, including in China. In the context of increasing digitalization, college students are deeply engaged with online and mobile technologies, which may also exacerbate digital inequalities. The second digital divide, conceptualized in this study as disparities in internet literacy and internet attitudes after basic access to digital technologies has been achieved, has emerged as an important form of digital inequality among college students. Its potential triggers and underlying mechanisms linking it to mental health remain underexplored. Parental rearing styles are established consistently linked to factors for depression and anxiety, but whether they affect mental health through the digital divide is unknown. This study aimed to (1) identify distinct parental rearing profiles; (2) evaluate the impact of different parental rearing styles on mental health issues; (3) investigate whether the second digital divide mediates this association.

**Methods:**

A cross-sectional study with a sample of 3533 college students was carried out. Latent profile analysis and multivariate logistic regression were conducted. Mediation analyses evaluated the mediating effects.

**Results:**

Three parental rearing style profiles were identified: positive, mixed, and negative parenting. Students with negative parental rearing style had the highest odds of depression, anxiety and their comorbidity, whereas those with mixed parental rearing styles also had higher odds than those with positive parental rearing styles. The second digital divide significantly mediated all associations. The subgroup and sensitivity analysis found that results remained unchanged.

**Conclusions:**

Narrowing the second digital divide and promoting positive rearing styles may serve as a valuable direction for college students’ mental health management.

## 1 Introduction

In the context of increasing digitalization, college students are deeply engaged with online and mobile technologies, which bring both benefits and inequalities (1). Digitalization-related inequality may act as a trigger for psychological stress (2). The prevalence of depression and anxiety among students in higher education is on the rise globally (3). This global trend is also alarming in China. Recent systematic reviews found a prevalence of depression of 28.4% in Chinese college students (4), while a meta-analysis estimated the prevalence of anxiety symptoms among Chinese college students to be 25.0% (5). These psychological issues can adversely affect academic and career development, leading to problems such as reduced class attendance, lower academic performance, and increased social withdrawal (6), and higher risks of dropout and unemployment rate (2). Thus, understanding and addressing mental health during young adulthood is of vital public health importance.

Growing digital inequality is still a serious public health concern, despite the increasing digitalization among college students (7). Beyond its direct psychological impact, early life environment represented by parental rearing style plays a fundamental role in shaping individuals’ cognitive and behavioral patterns (8). Individuals’ digital literacy and attitudes in emerging adulthood is further influenced by these early formative experiences, which have a significant impact on their digital literacy and attitudes throughout college (9). Parental rearing style is therefore closely associated with the second digital divide (10). Specifically, authoritative parenting, which emphasizes warmth and appropriate autonomy, encourages children to use digital tools more independently. This independent engagement can foster hands-on experience and positive attitudes toward technology. Conversely, authoritarian parental rearing restricts autonomous access to digital tools, thereby reducing such learning opportunities, hindering digital skill development, and reinforcing negative views on technology use(Shi et al., 2026).

Beyond its role in shaping digital behaviors, parental rearing style is also a well-established contributor to mental health issues (11). Supportive and flexible parental rearing styles are positively associated with higher self-esteem and a reduced risk of mental health issues (12, 13), whereas negative or dysfunctional parenting practices have been identified as major risk factors for various psychiatric conditions, including depression, and anxiety and their comorbid symptoms (14, 15). Previous research has also shown that lower levels of digital integration are associated with more severe mental health issues, including higher rates of anxiety and depression (16). Given that parental rearing style influences both the second digital divide and mental health directly, it is plausible that the second digital divide serves as a mediator between parental rearing style and college students’ mental health. However, few studies have examined whether the second digital divide mediates the association between parenting styles and college students’ mental health.

### 1.1 The association between parental rearing style and mental health among college students

Traditionally, studies examining the link between parental rearing styles and mental health issues, such as depression and anxiety, have employed variable-centered approaches. However, these methods often neglect individual heterogeneity (12). Unlike variable-centered approaches that assume consistent variable associations across individuals, Latent Profile Analysis (LPA), as a person-centered technique, identify natural subgroups based on the clustering of different parenting dimensions (e.g., warmth, rejection, overprotection). This is particularly valuable for parenting research, as parents often show combinations of rearing behaviors that variable-centered methods cannot detect (17). Latent Profile Analysis (LPA), as a person-centered technique, enables the identification of distinct rearing style profiles, such as profiles characterized by high overprotection or rejection (17). This approach facilitates the classification of students into meaningful subgroups, thereby informing more precise and group-specific intervention strategies. A few previous studies have used latent profile analysis (LPA) to explore parental rearing style patterns (18). For example, one study identified two maternal parental rearing profiles in early childhood: low human stimulation (HS) & low social support (SS) and high HS & high SS group, which were found to differ significantly in children’s social-emotional development (19). Notably, current research on parental rearing styles and mental health issues has paid little attention to the comorbidity of depression and anxiety, especially lacking systematic analysis focusing on college students. the co-occurrence of depression and anxiety is associated with poorer clinical course trajectories and higher non-response rates compared to either symptoms alone (20). Our study aims to deepen previous study by using latent profile analysis to identify latent types of parental rearing styles, aiming to reveal the association between different parental rearing combinations and multiple psychological issues, including their comorbidity, among the study population of college students in digitalization context, and to provide theoretical basis and practical reference for precise psychological intervention.

### 1.2 The mediator of the second digital divide between the association of parental rearing style and mental health issues

The concept of the “digital divide” has evolved from a simple access issue to a multi-dimensional construct. The first-level digital divide traditionally refers to disparities in physical access to digital devices and the internet (21). The second-level divide captures inequalities in usage patterns including digital literacy and attitudes (22). The third-level divide concerns disparities in tangible outcomes and benefits derived from technology use (22, 23). Recent empirical data on Chinese college students indicate that although the first-level digital divide has considerably narrowed, access does not guarantee effective use (24, 25). China-specific studies reveal significant variation in digital literacy, search strategies, and tangible benefits among students—key contributors to the second- and third-level digital divides (24). Students from higher socioeconomic backgrounds tend to gain more from internet use, suggesting that online experiences may reinforce offline inequalities (24).

According to a systematic review (26), the second digital divide is triggered by various factors, including socioeconomic status, personal attributes, social support, and technology type. Among these triggers, scholars have acknowledged the parental rearing style has significant influences on their children’s second digital divide (27–29). More specifically, parental rearing style, typically characterized along dimensions of warmth and control, plays an essential role in shaping the digital usage patterns, which are key components of the second digital divide (30, 31). In contrast, authoritarian parenting, which emphasizes control with low emotional responsiveness, often restricts children’s autonomous access to digital tools, limiting hands-on experience. This may prevent children from developing practical digital skills, thereby widening the second-level divide despite access to technology (29).

The digital divide may give rise to many detrimental consequences, one of the major negative effects of the digital divide could be on one’s physical and mental well-being (32, 33). In the era of digital healthcare, the digital divide may prevent individuals from accessing digital health services (34). This exclusion reduces their ability to benefit from services such as teletherapy, mental health apps, and online support systems (35), which ultimately affects their physical and mental health. Based on the literature review, we proposed the following hypotheses:

H1. Several distinct parental rearing style profiles would be identified by latent profile analysis.
H2. Social and demographic characteristics (e.g., health status, subjective family social status and so on) will significantly distinguish latent parental rearing style profiles.
H3. It is hypothesized that the association between parental rearing styles and mental health issues (depression, anxiety, and their comorbidity) may be partially explained by the second digital divide. Specifically, positive parental rearing styles are expected to be associated with a lower level of the second digital divide (i.e., higher levels of internet literacy and more positive internet attitudes), which in turn may be associated with lower levels of depression, anxiety, and depression–anxiety comorbidity.

**Figure 1:**
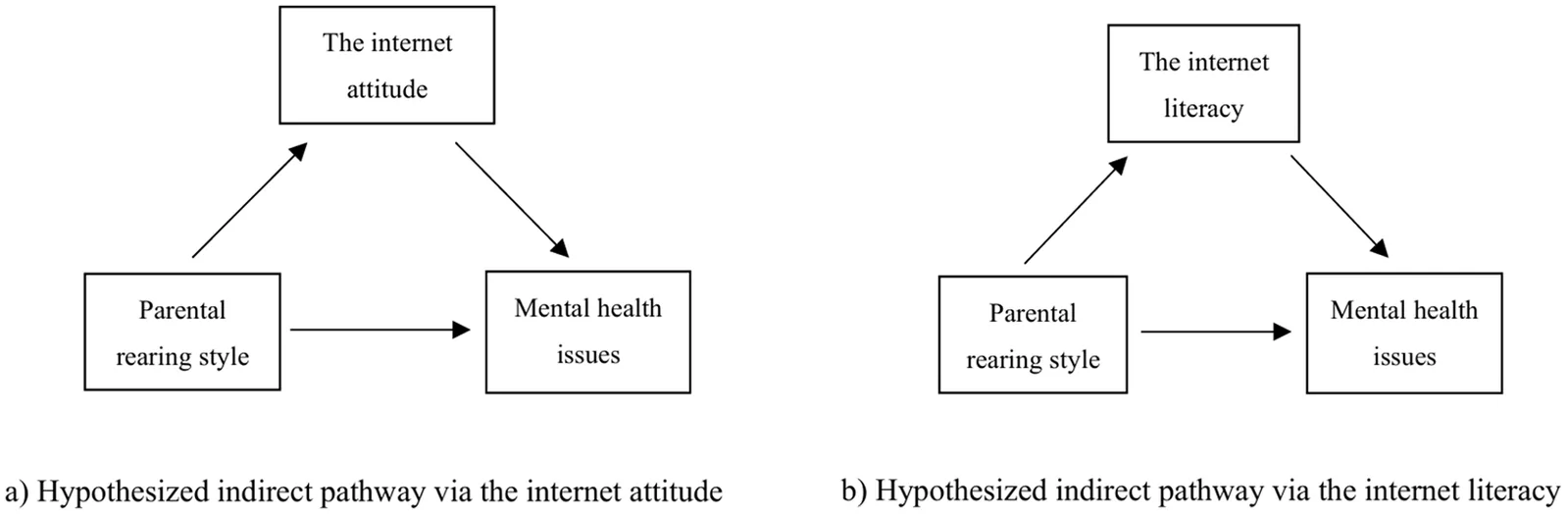
The hypothesized indirect pathway model of the association between parental rearing style and mental health issues (depression, anxiety, and their comorbid symptoms) among college students, mediated by **(a)** internet attitude and **(b)** internet literacy.

The hypotheses model is shown in **Figure 1**.

## 2 Methods

### 2.1 Participants and procedure

#### 2.1.1 Participants

A cross-sectional survey was conducted among college students in China as part of a larger longitudinal projection on adolescent behavior, cognition and mental health from May 2024 to August 2024. The study participants were college students in four universities in Hubei province of China. The four universities included two double first-class universities and two ordinary universities, which were selected based on their willingness to participate in the study and to allow the research team to conduct data collection. To protect institutional confidentiality and comply with the data collection agreements established with participating universities, university identifiers were not recorded in the final survey dataset. Therefore, university-level clustering analyses could not be conducted. The inclusion criteria of participants were: (1) age 18 years or older; (2) currently enrolled as an undergraduate student at one of the participating universities; and (3) ability to understand and complete the questionnaire independently.

Participants were recruited through voluntary participation at the sampled universities, with each participant entering a lottery for a small gift (valued at approximately 10 RMB) upon completion of the questionnaire. To ensure response validity, an attention check was embedded in the questionnaire using reverse-coded items. Specifically, pairs of positively and negatively worded questions measuring the same construct were presented. Participants who provided contradictory responses to these paired items (e.g., agreeing with both the positive and negative statements of the same content) were considered inattentive and excluded from the final sample. A total of 3,997 students were approached. After excluding those who failed the attention check (n = 211) and those with incomplete data (n = 253), the final analytic sample consisted of 3,533 college students.

#### 2.1.2 Procedure

The survey was administered via an online self-administered and anonymous questionnaire. All scales used in this study were previously validated Chinese versions that have been widely applied among Chinese populations. Teachers at the sampled universities distributed the questionnaire link and QR code in class groups and invited students to complete the survey voluntarily. To guarantee smooth participation and high-quality responses, research assistants were assigned to each university to provide online Q&A support throughout the data collection period. All participants completed the questionnaire online on an anonymous basis.

### 2.2 Measures

#### 2.2.1 Sociodemographic information form

The sociodemographic information form includes a range of demographic variables reported by the participants themselves, such as gender, age, grade, and academic major and so on.

#### 2.2.2 Parental rearing style

Parental rearing style was measured by a short form of Chinese version of Egna Minnen av Barndoms Uppfostran (EMBU) scale (36, 37). There were 21 items to evaluate the parental rearing style (6 items for rejection, 7 items for warmth, and 8 items for overprotection). Regarding the reliability of the instruments, the Chinese version of the short-form EMBU (s-EMBU-C) was validated in a previous study (36), which reported Cronbach’s α coefficients ranging from 0.74 to 0.84 across the three subscales (rejection, emotional warmth, and overprotection) for both the paternal and maternal forms. In our study, the Cronbach’s α coefficients for each scale were as follows: 0.93,0.94,0.81 for maternal rejection, warmth, and overprotection, respectively; 0.92,0.94,0.82 for paternal rejection, warmth, and overprotection, respectively.

The items are scored on a five-point Likert scale (1: never- 5: always). These six continuous variables served as the indicator variables for the LPA.

#### 2.2.3 Depression

Given that the present study focused specifically on depression and anxiety symptoms and their comorbidity as the primary mental health outcomes, only the depression and anxiety subscales of the DASS-21 were administered. The depression subscale of Chinese version of Depression Anxiety Stress Scale-21 was used to evaluate depression symptoms (38, 39), with 7 items in total. The Chinese version of the DASS-21 was validated in a previous study (39), which reported Cronbach’s α of 0.77 for the depression subscale. In this study, the Cronbach’s α coefficient for subscale is 0.92. Each item has a score of 0 (not at all)–3 (almost every day), with a total score of 0–21. A total (final scores need to be multiplied by 2) score exceeding 9 is considered abnormal, indicating depression.

#### 2.2.4 Anxiety

The anxiety subscale of Chinese version of Depression Anxiety Stress Scale-21 was used to evaluate anxiety symptoms (38, 39), with 7 items in total. The Chinese version of the DASS-21 was validated in a previous study (39), which reported Cronbach’s α of 0.79 for the anxiety subscale. In this study, the Cronbach’s α coefficient for subscale is 0.91. Each item has a score of 0 (not at all) –3 (almost every day), with a total score of 0–21. A total (final scores need to be multiplied by 2) score exceeding 7 is considered abnormal, indicating anxiety.

#### 2.2.5 The second digital divide

In this study, the second digital divide was conceptualized as an individual-level construct reflecting disparities in internet literacy and internet attitudes after basic access to digital technologies has been achieved, which is also referred to as the usage divide (26). This construct focuses on how well individuals use digital technologies rather than whether they merely have access to them. Consistent with previous studies (33, 40, 41), the construct was operationalized through two dimensions: internet literacy and internet attitude. Internet literacy was measured using five items evaluating skills in search engine use, database retrieval, information evaluation, software operation, and programming (scores ranging from 1 = very low to 5 = very high, reflecting higher proficiency) (33, 41). A weighted composite score was calculated based on each skill’s academic relevance (weights: 15%, 25%, 25%, 15%, and 20%, respectively) (42), with higher scores indicating higher levels of internet literacy. Internet attitude was measured using three items assessing respondents’ attitudes toward internet use (25, 33), with higher scores indicating more positive attitudes toward internet use. Thus, higher scores indicated higher levels of internet literacy and more positive internet attitudes, indicating lower vulnerability to the second digital divide. In our study, the Cronbach’s α coefficients of internet literacy subscale and internet attitude subscale were respectively 0.86 and 0.91.

#### 2.2.6 Statistical analysis

##### 2.2.6.1 Descriptive analyses

Descriptive statistics (i.e., mean, standard deviation, or frequency) were used to summarize the sample characteristics. Differences of the background characteristics of college students were assessed by one-way analysis of variance (ANOVA) for continuous variables and chi-square test for categorical variables. Latent profile analysis (LPA) was used to determine the patterns of parental rearing style. Six continuous variables were used as indicators in the latent profile analysis, including paternal emotional warmth, paternal rejection, paternal overprotection, maternal emotional warmth, maternal rejection, and maternal overprotection. The 21 EMBU items were grouped into these six dimensions, and scores for each dimension were calculated by averaging the corresponding items, with higher scores indicating higher levels of the corresponding parenting characteristic. Since all indicators were measured on the same 1–5 Likert scale, the original scores were directly entered into the latent profile analysis without standardization. Fit indices were estimated for the LPA models for 1-class through 4-class solutions. We selected the best-fitting class solution using the following criteria: 1) lower values on Akaike Information Criterion (AIC), Bayesian Information Criterion (BIC), and adjusted Bayesian Information Criterion (aBIC); 2)Entropy > 0.70, values closer to 1 indicate more accurate classification; 3) at least 5.0% of participants should be classified into each identified class (43); 4)significant LMR and BLRT p-values (p < 0.01) indicating the better fit of a k-profile model compared to a k-1 profile model (44).

##### 2.2.6.2 Primary analyses

Multivariable logistic regression was used to examine the association between different profiles of parental rearing style and mental health issues among college students and reported unadjusted and adjusted odds ratios (OR) and 95% confidence intervals (95% CI). For each mental health outcome, three logistic regression models were fitted with the positive parental rearing style profile as the reference category. Model 1 (crude model) included only the parental rearing style profile variable. Model 2 additionally adjusted for sociodemographic variables (social status, gender, age, poverty, and health status). Model 3 additionally adjusted for academic performance variables (rank range, grade, and major) based on Model 2. Cases with missing data on any variable were excluded using listwise deletion. Clustering by university was not considered because all four universities are located in the same province with comparable academic standards and student populations, and no substantial between-university heterogeneity was expected.

##### 2.2.6.3 Mediation analyses

We conducted mediation analyses to investigate the mediating role of the second digital divide on the association between different profiles of parental rearing style and mental health problems including depression, anxiety and depression-anxiety comorbidity. Uncertainty was assessed using bias-corrected bootstrapping with 5,000 resamples to generate 95% confidence intervals for indirect effects. We conducted mediation analysis using the mediation package in R (45). In this mediation analysis, the three-category parental rearing profile was treated as a nominal categorical variable and dummy-coded with the positive parental rearing profile as the reference group. All effect sizes reported below are standardized regression coefficients (β). We estimated the overall natural direct effect (NDE) and natural indirect effect (NIE) across the full sample, rather than separate effects for pairwise group contrasts, as our primary research focus lies in the general mediating mechanism linking parental rearing profiles to mental health outcomes. All effect sizes reported below are standardized regression coefficients (β).

##### 2.2.6.4 Stratified analyses

We repeated models stratified by college students’ gender (categorized as male or female). Results from these stratified analyses are reported descriptively.

##### 2.2.6.5 Sensitivity analyses

To control for potential confounding, we conducted two sensitivity analyses: (1) restricting the sample to the normal college age range (18–23 years) to exclude atypical educational trajectories; and (2) including only participants with higher perceived social status(the score of social status not less than 4, on a 1–5 scale) to test whether the association holds in a subgroup with less social disadvantage, which would argue against confounding by social status.

All statistical analyses were performed using R version 4.2.1 and Mac. Mplus software (version 8.3), and statistical significance was ascertained by a two-sided P value < 0.05.

## 3 Result

### 3.1 Descriptive statistics

A total of 3,997 students were approached. After excluding those who failed the attention check (n = 211) and those with incomplete data (n = 253), the final analytic sample consisted of 3,533 college students (valid response rate = 88.4%). Among the 3,533 participants, the majority were female (76.39%) and aged between 18 and 23 years (75.77%). Statistically significant differences between groups (e.g., by gender, by health status and so on) were observed across all categorical and continuous variables (see **Table 1**, **Supplementary Table 1** for detailed comparisons).

**Table 1:** Characteristics of the study sample(N = 3533).

Variables	Categories	N	Percentage (%)	χ^2^-tests
Gender	Male	834	23.61%	984.50^***^
	Female	2699	76.39%	
Rank range	Top 10%	619	17.52%	740.21^***^
	11% – 25%	881	24.94%	
	26% – 50%	1206	34.14%	
	51% – 75%	592	16.76%	
	Bottom 25%	235	6.65%	
Age (in years)	≤18	76	2.15%	7041.48^***^
	18-23	2677	75.77%	
	24-29	513	14.52%	
	30-35	156	4.42%	
	≥36	111	3.14%	
Subjective family social status	Bottom	165	4.66%	4280.15^***^
Subjective family social status	Low	628	17.78%	
	Middle	2219	62.81%	
	High	401	11.35%	
	Top	120	3.40%	
Health status	very poor	312	8.83%	543.06 ^***^
	Poor	1100	31.14%	
	Normal	708	20.04%	
	Good	776	21.96%	
	Excellent	637	18.03%	
Poverty	Yes	1093	30.94%	513.56^***^
	No	2440	69.06%	
Major	Arts and Humanities	1442	40.82%	94.42^***^
	Medicine	1107	31.33%	
Major	Science Others	492 492	13.93% 13.93%	
Grade	Freshman	262	7.42%	1945.28^***^
	Sophomore	1613	45.66%	
	Junior	1459	41.30%	
	Senior	199	5.63%	
Depression	Yes	1092	30.91%	515.09^***^
	No	2441	69.09%	
Anxiety	Yes	1346	38.10%	200.19^***^
	No	2187	61.90%	
Comorbidity	Yes	963	27.26%	730.95^***^
	No	2570	72.74%	
Variables	Continuous	Mean	SD	t-tests
Study hours per week	/	25.46	18.89	78.81^***^
Internet hours per week	/	25.49	18.97	73.59^***^

***P<0.001, χ^2^-tests were used for categorical variables, and t-test was used for continuous variables.

### 3.2 Common method bias

The Harman single factor method results showed that there were 10 factors with a characteristic of value greater than 1, and the first factor only explained a variation of 33.10%, which was much less than the 40% critical value (46). These results suggest that common method bias is unlikely to be a major threat to the validity of our findings.

### 3.3 Latent profile analysis of parental rearing style

**Figure 2:**
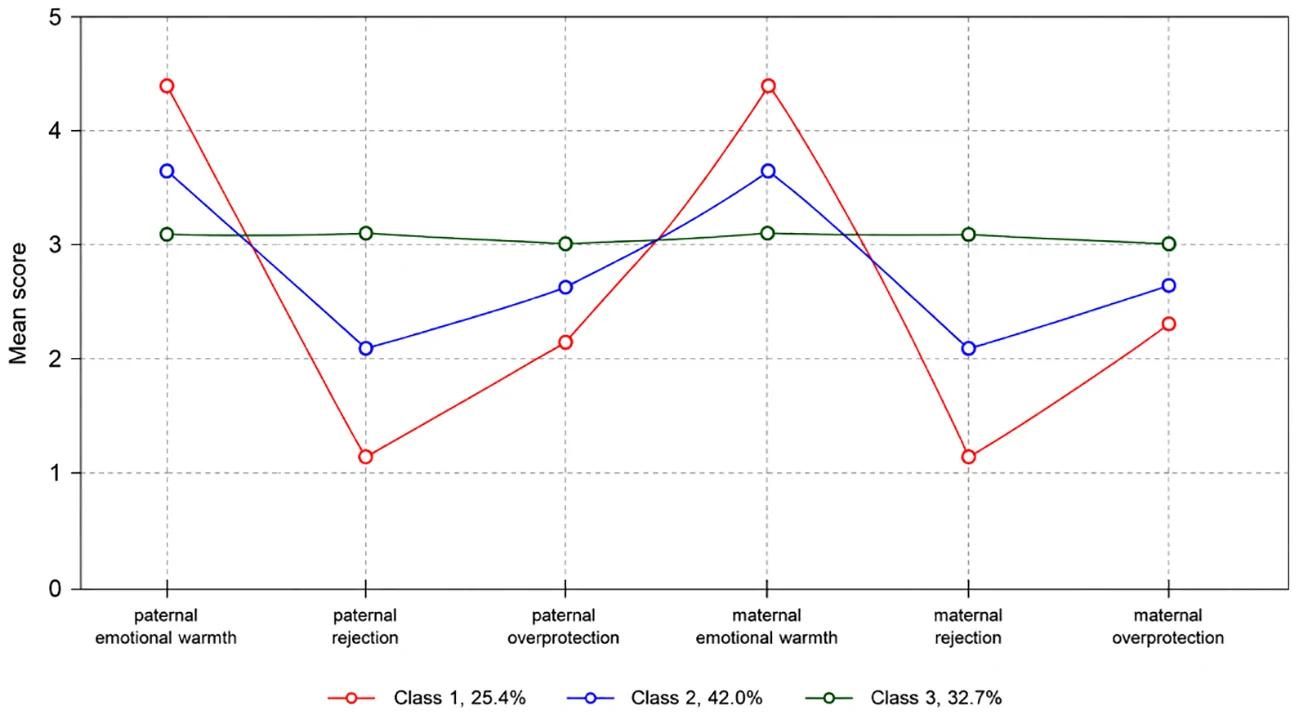
Classes identified in the total sample through latent profile analysis. Note:class 1: positive rearing style; class 2: mixed rearing style; class 3: negative rearing style.

Based on model fit and interpretability, a three-profile solution was selected (**Table 2**, **Figure 2**). Although the 4-profile solution yielded slightly better information criteria (e.g., lower AIC and BIC) compared to the 3-profile solution, one of the four profiles contained fewer than 5% of the sample (n < 5% threshold), which we considered insufficient for meaningful subsequent analysis (47). The three classes were labeled as positive, mixed, and negative rearing styles based on emotional warmth, rejection, and overprotection scores. Sample sizes and mental health issues for each class are summarized in **Table 3**. The prevalence of depression, anxiety, and comorbidity increased substantially from positive to negative profiles, indicating strong associations between parental rearing styles and mental health issues.

**Table 2:** Fit indices, entropy, and model comparisons for estimated latent profile analyses models.

Model	AIC	BIC	SSA-BIC	Entropy	pLMR	pBLRT	Group size for each profile			
							1	2	3	4
1-class	49431.33	49505.37	49467.24	/		/	3533(100.00%)			
2-class	42952.37	43069.60	43009.23	0.846	<0.001	<0.001	1933(54.71%)	1600(45.29%)		
3-class	39433.45	39593.86	39511.25	0.946	<0.001	<0.001	896(25.36%)	1489(42.15%)	1148(32.49%)	
4-class	37157.68	37361.28	37256.43	0.933	<0.001	<0.001	1434(40.59%)	1087(30.77%)	903(25.56%)	109(3.09%)

AIC, Akaike information criterion; BIC, Bayesian Information Criterion; SSA-BIC, Sample Size Adjusted Bayesian Information Criterion; Adjusted LMR test, Adjusted Lo-Mendell-Rubin likelihood ratio test; BLRT, Bootstrapped likelihood ratio test. Bold corresponds to the model with the best fit.

**Table 3:** Logistic regression examining the association between different profiles of parental rearing styles and mental health issues among college students(N = 3533).

Depression(N = 1092)	No. of exposed cases	Model 1 OR (95% CI)	Model 2 OR (95% CI)	Model 3 OR (95% CI)
Class 1	101	Ref.	Ref.	Ref.
Class 2 vs Class 1	292	1.92(1.51,2.45)	1.92(1.51,2.46)	1.92(1.51,2.47)
Class 3 vs Class 1	699	12.25(9.69,15.63)	11.97(9.43,15.33)	11.89(9.36,15.23)
Anxiety(N = 1346)				
Class 1	144	Ref.	Ref.	Ref.
Class 2 vs Class 1	447	2.24(1.82, 2.77)	2.16(1.75, 2.68)	2.19(1.76,2.71)
Class 3 vs Class 1	755	10.03(8.10,12.49)	9.44(7.60, 11.79)	9.50(7.46,11.88)
Comorbidity(N = 963)				
Class 1	78	Ref.	Ref.	Ref.
Class 2 vs Class 1	241	2.03(1.55,2.66)	2.01(1.53,2.65)	2.02(1.55,2.66)
Class 3 vs Class 1	644	13.40(10.39,17.49)	13.14(10.15,17.21)	13.13(10.13,17.22)

OR, odds ratio; CI, confidence interval. Model 1: crude analysis. Model 2: adjusted for sociodemographic variables, with regard to social status, gender, age, poverty and health status based on model 1, Model 3: adjusted for academic performance variables, with regard to rank range, grade, major based model 1. Ref: Reference group.

### 3.4 Associations between parental rearing style profiles and college students’ depression, anxiety and their comorbidity

As shown in **Supplementary Table 3**, χ**^2^**-tests revealed significant differences across the three parental rearing style groups in terms of academic ranking, subjective family socioeconomic status, self-rated health, age, academic major, and gender. Participants in Class 1 were more likely to report favorable issues across multiple domains. **Table 3** presents the associations between different profiles of parental rearing styles and mental health issues among college students, including depression, anxiety, and comorbid symptoms. Class 1 served as the reference group in all models. In the final adjusted model (Model 3), compared to college students exposed to positive rearing style (Class 1), those exposed to the mixed rearing style (Class 2) had a significantly increased odds of depression (OR = 1.92, 95% CI: [1.51–2.47]), anxiety (OR = 2.19, 95% CI: [1.76–2.71]), and comorbid anxiety and depression (OR = 2.02, 95% CI: [1.55–2.66]). College students exposed to a negative rearing style (Class 3) had the highest odds for all three mental health issues. Specifically, college students exposed to a negative rearing style had 11.89 times the odds of depression (OR = 11.89, 95% CI: [9.36–15.23]), 9.50 times the odds of anxiety (OR = 9.50, 95% CI: [7.46–11.88]), and 13.13 times the odds of comorbid anxiety and depression (OR = 13.13, 95% CI: [10.13–17.22]) compared to those exposed to a positive rearing style. These associations remained robust after controlling for potential confounders across all three models.

### 3.5 Mediation analysis

#### 3.5.1 Internet attitude divide

Analysis of statistical indirect pathways showed that internet attitude acted as a partial intermediate factor in the associations between parental rearing profiles and mental health issues. For depression, the adjusted natural indirect effect (NIE) was 0.02 (95% CI: 0.01–0.03), and the natural direct effect (NDE) was 0.07 (95% CI: 0.05–0.10). For anxiety, the NIE was 0.01 (95% CI: 0.01–0.02) and the NDE was 0.12 (95% CI: 0.08–0.16). For comorbid depression and anxiety, the NIE was 0.01 (95% CI: 0.01–0.02) and the NDE was 0.12 (95% CI: 0.08–0.16). These results indicate that internet attitude represents a small yet statistically significant intermediate factor linking parental rearing styles to psychological distress (**Table 4**).

**Table 4:** Total, direct and indirect effects of mediators, internet attitude and internet literacy for the association between parental rearing style and mental health problems among college students(N = 3533)^a,b^.

	Total effect (β,95% CI)	Natural direct effect (β,95% CI)	Natural indirect effect (β,95% CI)	Mediation percentage (%,95% CI)
Depression(N = 1092)				
Mediator: internet attitude	0.09(0.06,0.12)	0.07(0.05,0.10)	0.02(0.01,0.03)	22.22(8.87,35.57)
Mediator: internet literacy	0.09(0.07,0.11)	0.07(0.05,0.10)	0.01(0.01,0.02)	11.11(5.03,17.19)
Anxiety(N = 1346)				
Mediator: internet attitude	0.13(0.09,0.17)	0.12(0.08,0.16)	0.01(0.01,0.02)	7.69 (3.18,12.20)
Mediator: internet literacy	0.14(0.11,0.17)	0.11(0.09,0.15)	0.02(0.02,0.03)	14.29(9.59,18.99)
Comorbidity(N = 963)				
Mediator: internet attitude	0.08(0.04,0.12)	0.07(0.04,0.10)	0.01(0.01,0.02)	12.50(3.66, 21.34)
Mediator: internet literacy	0.08(0.06,0.10)	0.07(0.05,0.09)	0.01(0.01,0.02)	12.50 (5.52,19.48)

a. All outcomes are binary (e.g. depression vs no depression), where positive parental rearing style is the reference group.

b. Adjusted for exposure–mediator, mediator–outcome and exposure–outcome confounders (academic performance and social and demographic background factors) as listed in **Table 4**.

c. Effect values are standardized regression coefficients (β).

d. Mediation percentage = (Natural indirect effect/Natural total effect) × 100%.

#### 3.5.2 Internet literacy divide

Internet literacy also functioned as a partial intermediate factor in the observed associations. For depression, the adjusted NIE was 0.01 (95% CI: 0.01–0.02) and the NDE was 0.07 (95% CI: 0.05–0.10). For anxiety, the NIE was 0.02 (95% CI: 0.02–0.03) and the NDE was 0.11 (95% CI: 0.09–0.15). For comorbid symptoms, the NIE was 0.01 (95% CI: 0.01–0.02) and the NDE was 0.07 (95% CI: 0.05–0.09). Overall, internet literacy served as a modest but significant intermediate variable in the associations between parental rearing styles and psychological distress (**Table 4**).

#### 3.5.3 Stratified and sensitivity analysis

No significant interaction effects by gender were found (p > 0.05 for all outcomes), indicating that the observed associations did not differ between male and female students. Sex-stratified analyses yielded consistent results: mixed and negative parental rearing profiles were linked to a greater likelihood of reporting mental health symptoms compared with positive parental rearing style (**Supplementary Tables 5**, **6**). Confidence intervals largely overlapped across gender subgroups, further supporting consistent patterns. Stratified indirect pathway analyses also showed no meaningful differences between sexes (**Supplementary Tables 7**, **8**). Sensitivity analyses restricted to specific age ranges and levels of perceived social status did not alter the overall findings (**Supplementary Tables 9**-**12**).

## 4 Discussion

Our study finds that nearly 30.91%,38.10%, and 27.26% of the college students showed depression, anxiety and their comorbid symptoms, suggesting that mental health difficulties affect around one-third of participating college students. The prevalence of depression under positive, mixed, and negative parental rearing styles was 11.27%, 19.61%, and 60.89%, respectively. Corresponding rates for anxiety were 16.07%, 30.02%, and 65.77%, and for comorbidity were 8.71%, 16.19%, and 56.10%. These results show a distinct gradient, with students exposed to negative parental rearing styles having the highest prevalence of mental health issues, followed by mixed group and positive group. It should be noted that there was a substantial gender imbalance in our sample, with 23.61% male and 76.39% female participants. This pattern is commonly observed in mental health research, as females tend to show greater interest in and willingness to participate in health-related surveys (48, 49). More importantly, this imbalance also reflects the demographic composition of the specific universities from which the sample was drawn, as female students naturally constitute a larger share of the student population in these institutions. Importantly, subgroup analyses and sensitivity tests confirmed that the observed associations remained stable across different gender groups, suggesting that the gender imbalance did not substantively affect the robustness of our primary findings.

This finding is consistent with the commonly held view that less positive parental rearing styles are associated with poorer mental health (50, 51). Notably, the mixed parental rearing profile consistently showed intermediate levels of depression, anxiety, and comorbidity between the positive and negative profiles. This finding suggests that parental behaviors may not be uniformly positive or negative but rather reflect a combination of supportive and adverse characteristics. The relatively high prevalence of this profile may indicate that many Chinese families simultaneously exhibit parental warmth and behavioral control, a pattern that has frequently been described in East Asian parenting contexts (52, 53).

Consistent with previous research, our findings confirm a significant relationship between parental rearing styles and mental health issues among Chinese college students (54). We found that children under negative parental rearing style were more likely to be suffering from depression, anxiety and their comorbidity (37). Positive parenting, characterized by high warmth and low overprotection or rejection, appears to serve as a protective factor against mental health problems. Previous research has identified this parental rearing style as an appropriate and beneficial combination for child development (55). However, another previous study reported that permissive parental rearing style (high warmth and low strictness, similar to positive parenting) tends to be related to negative developmental outcomes for adolescents (56). These inconsistencies may arise from methodological and classification variations across studies. Additionally, since data rely on offspring’s reports, it can be difficult for them to clearly distinguish positive parental rearing style from permissive styles.

The second digital divide, operationalized through digital literacy and digital attitudes, showed a potential indirect pathway linking parental rearing styles to mental health issues including depression, anxiety, and comorbidity, which provides evidence supporting Hypothesis 3. These associations remained significant after adjusting for academic performance and sociodemographic covariates and were stable in subgroup analyses and sensitivity tests after excluding students with abnormal entry age and those who perceived themselves as not having higher social status. Consistent with prior research on adults, our study confirms the digital divide’s relevance to the mental health of college students (34, 57). Prior research on adults has established that digital access problems, which are often regarded as the first digital divide, are associated with adverse mental health issues (57). However, apart from access-related issues, digital inequality also involves two other dimensions of the second digital divide: digital skills and digital attitudes. A cross-sectional study of 1500 Chinese college students found that e-health literacy, a core dimension of the second digital divide, partially mediated the association between self-efficacy and depressive symptoms (58). A systematic review of 32 studies further confirmed that higher digital health literacy is consistently associated with better mental health outcomes, including lower levels of depression, anxiety, and loneliness, as well as higher well-being and quality of life, among Chinese college students (59). In addition, a randomized controlled trial of 93 stressed university students demonstrated that digital attitudes significantly reduced hopelessness and trait anxiety (60). Prior research also indicates that despite equal access to technology, certain parental practices may hinder the development of practical digital skills, contributing to the second digital divide (27–29). Notably, inconsistencies in prior findings may stem from varying definitions and measures.

Beyond the observed association, several mechanisms may explain how parental rearing styles contribute to the second digital divide. Negative parental rearing styles, characterized by high levels of rejection and overprotection, may limit opportunities for independent exploration, problem-solving, and autonomous learning. Such family environments may hinder the development of self-efficacy and confidence in using digital technologies, resulting in lower digital literacy and less positive digital attitudes (61, 62). In contrast, positive parental rearing styles may encourage autonomy, curiosity, and active engagement with information resources, thereby facilitating the acquisition of digital competencies (61, 62). These developmental influences may persist into young adulthood and contribute to disparities in digital literacy and digital attitudes even when access to digital technologies is relatively equal (61).

The association between the second digital divide and mental health may also be understood through several potential pathways. Students with lower digital literacy may experience greater difficulty in locating reliable health information, accessing online psychological support, or effectively utilizing digital educational resources (59). Furthermore, limited digital competence may increase frustration, information overload, and feelings of exclusion in increasingly digitalized academic environments (34, 57). Previous studies have suggested that higher levels of digital literacy facilitate access to health information and coping resources and are associated with lower levels of depressive and anxiety symptoms (58, 59). In addition, positive digital attitudes may promote engagement with beneficial online services and support systems, thereby reducing psychological distress (60).

From a theoretical perspective, this study extends existing research on parental influences and mental health by identifying the second digital divide as a potential explanatory pathway linking parental rearing styles to depression, anxiety, and their comorbidity. Previous studies have largely focused on the direct associations between family environments and psychological outcomes (61, 62). Our findings suggest that digital literacy and digital attitudes may represent an important developmental mechanism through which early family experiences continue to influence mental health in the digital era. By identifying three distinct parental rearing profiles in a large Chinese college sample, this study also moves beyond variable-centered approaches to reveal heterogeneous patterns of parenting experiences. Clinically, these findings highlight the importance of screening students from negative or mixed rearing backgrounds for early intervention and suggest that improving digital literacy and fostering positive digital attitudes may offer a practical and modifiable target for mental health promotion on college campuses. Socially, the results underscore the need for universities to integrate digital literacy education into their curricula and for policymakers to address digital inequality as part of broader efforts to reduce mental health disparities among college students.

## 5 Conclusion and limitation

Three parental rearing profiles (positive, mixed, and negative) were identified among college students. Negative parental rearing was linked to higher prevalence of depression, anxiety and their comorbidity, whereas positive parental rearing style correlated with lower symptom rates. As parenting patterns tend to be stable and difficult to modify within established family dynamics, the second digital divide s may represent a potentially important intermediate factor in the observed associations between parental rearing style and mental health. Narrowing the second digital divide and promoting positive rearing styles may serve as a notable direction for college students’ mental health management.

This study has several limitations. First, considering the cross-sectional design and self-reported data sources, the present findings cannot establish definite causal directions. Specifically, current mental health status may bias retrospective recollection of parental rearing styles and subjective evaluation of individual digital competence, and unmeasured confounding variables cannot be fully eliminated. The sample’s restriction to Chinese university students also limits generalizability. In addition, our sample had a substantial gender imbalance with 76.39% female participants. Subgroup analyses and sensitivity tests confirmed that the observed associations remained stable across gender groups, which indicated that this imbalance did not substantially compromise the robustness of our primary findings. We still suggest future research adopt gender-balanced samples to verify our conclusions. Longitudinal studies are required in the future. Second, university identifiers were not retained in the final dataset due to institutional confidentiality requirements. Therefore, potential university-level clustering effects could not be examined in the present study. Third, although this procedure helped ensure participant anonymity and institutional privacy, future studies may further explore university-level influences using multi-center datasets with identifiable institutional information.

## Data Availability

The datasets presented in this article are not readily available because The data that support the findings of this study are available from [corresponding author, Jie Yu] but restrictions apply to the availability of these data, which were used under license for the current study, and so are not publicly available. Data are available upon reasonable request with permission of the corresponding author (Jie Yu) Requests to access the datasets should be directed to Jie Yu, jyu075@whu.edu.cn.
