# Supplementary material for: The second digital divide mediates the association between parental rearing style and depression, anxiety and their comorbidity among college students: a latent profile and mediation analysis
Source: Front Psychiatry. 2026 Aug 19;17:1838943. doi: 10.3389/fpsyt.2026.1838943 (PMC13534101; doi:10.3389/fpsyt.2026.1838943)
Supplement: Supplementary file 1 [file Table1.docx]

Supplementary Material

| **Variables** | **Mean** | **SD** | **anxiety** | **depression** | | **paternal overprotection** | | **maternal overprotection** | | **paternal rejection** | | **maternal rejection** | | **paternal warmth** | | **maternal warmth** | | |  |
| --- | --- | --- | --- | --- | --- | --- | --- | --- | --- | --- | --- | --- | --- | --- | --- | --- | --- | --- | --- |
| **Anxiety** | 1.1 | 0.86 | **0.88** | |  | |  | |  | |  | |  | |  | |  |  |  |
| **Depression** | 1.02 | 0.72 | 0.83^***^ | | **0.86** | |  | |  | |  | |  | |  | |  |  |  |
| **paternal overprotection** | 2.80 | 0.72 | 0.23 ^***^ | | 0.25^***^ | | **0.78** | |  | |  | |  | |  | |  |  |  |
| **maternal overprotection** | 2.82 | 0.73 | 0.22^***^ | | 0.23^***^ | | 0.77^***^ | | **0.78** | |  | |  | |  | |  |  |  |
| **paternal rejection** | 2.18 | 0.83 | 0.45^***^ | | 0.48^***^ | | 0.39^***^ | | 0.38^***^ | | **0.91** | |  | |  | |  |  |  |
| **maternal rejection** | 2.16 | 0.83 | 0.45^***^ | | 0.48^***^ | | 0.39^***^ | | 0.40^***^ | | 0.87^***^ | | **0.91** | |  | |  |  |  |
| **paternal warmth** | 3.74 | 0.77 | -0.39^***^ | | -0.42^***^ | | -0.06^***^ | | -0.06^***^ | | -0.57^***^ | | -0.54^***^ | | **0.87** | |  |  |  |
| **maternal warmth** | 3.81 | 0.76 | -0.40^***^ | | -0.43^***^ | | -0.08^***^ | | -0.06^***^ | | -0.53^***^ | | -0.56^***^ | | 0.85^***^ | | **0.87** |  |  |

**Table S1. Cronbach's α and correlations between all variables.**

**Note: *** p < 0.001,diagonal bold values are the square roots of the Average Variance Extracted (AVE).**

**Table S2. Between-class-differences i****n the parental dimensional variables used to identify the classes (N=3533).**

|  | **Class 1: Positive**  **(n=896 ,25.36%)** | **Class 2: Mix**  **(n =1489,42.15%)** | **Class 3: Negative**  **(n =1148,32.49%)** |  | | |
| --- | --- | --- | --- | --- | --- | --- |
| **Father** | M SD | M SD | M SD | F | p | *η*^2^ |
| **Rejection** | 1.11 0.20 | 2.08 0.28 | 3.13 0.46 | 706.52 | <0.001 | 0.02 |
| **Emotional warmth** | 4.38 0.79 | 3.81 0.54 | 3.15 0.55 | 324.21 | <0.001 | 0.02 |
| **Overprotection** | 2.39 0.85 | 2.83 0.64 | 3.10 0.55 | 6.04 | 0.03 | 0.17 |
| **Mother** |  |  |  |  |  |  |
| **Rejection** | 1.10 0.18 | 2.06 0.28 | 3.12 0.44 | 966.22 | <0.001 | 0.01 |
| **Emotional warmth** | 4.42 0.79 | 3.88 0.54 | 3.21 0.54 | 356.43 | <0.001 | 0.01 |
| **Overprotection** | 2.40 0.86 | 2.85 0.65 | 3.13 0.56 | 5.93 | 0.035 | 0.18 |
| **Participants above clinical thresholds** |  |  |  |  |  |  |
|  | N % | n % | n % | χ^2^ | p | Ф |
| **Depression** | 101 11.27% | 292 19.61% | 699 60.89% | 733.95 | <0.001 | 0.46 |
| **Anxiety** | 144 16.07% | 447 30.02% | 755 65.77% | 599.91 | <0.001 | 0.41 |
| **Comorbidity** | 78 8.71% | 241 16,19% | 644 56.10% | 729.17 | <0.001 | 0.45 |

**Note: M: mean, SD: standard deviation, N: number.**

**Table S3. Between-class-differences in** **academic performance and social and demographic background among different parental rearing profiles.**

|  | **Class 1: Positive parental rearing style(n=896)** | **Class 2: Mixture parental rearing style (n =1489)** | **Class 3: Negative rearing style (n =1148)** |  |  |  |
| --- | --- | --- | --- | --- | --- | --- |
|  | **n %** | **n %** | **n %** | **χ^2^** | **p** | **Ф** |
| **Rank range** |  |  |  | 40.99 | <0.001 | 0.11 |
| **Top 10%** | 190 21.21% | 245 16.45% | 184 16.02% |  |  |  |
| **11% – 25%** | 240 26.79% | 402 27.01% | 239 20.82% |  |  |  |
| **26% – 50%** | 276 30.80% | 511 34.31% | 419 36.50% |  |  |  |
| **51% – 75%** | 136 15.18% | 252 16.92% | 204 17.77% |  |  |  |
| **Bottom 25%** | 54 6.02% | 79 5.31% | 102 8.89% |  |  |  |
| **Subjective family social status** |  |  |  | 164.58 | <0.001 | 0.22 |
| **Bottom** | 41 4.58% | 44 2.95% | 80 6.97% |  |  |  |
| **Low** | 110 12.28% | 269 18.07% | 249 21.69% |  |  |  |
| **Middel** | 532 59.38% | 957 64.27% | 730 63.59% |  |  |  |
| **High** | 156 17.41% | 193 12.96% | 52 4.53% |  |  |  |
| **Top** | 57 6.35% | 26 1.75% | 37 3.22% |  |  |  |
| **Health status** |  |  |  | 157.55 | <0.001 | 0.21 |
| **very poor** | 71 7.92% | 139 9.34% | 102 8.89% |  |  |  |
| **Poor** | 286 31.92% | 503 33.78% | 311 27.09% |  |  |  |
| **Normal** | 109 12.17% | 259 17.39% | 340 29.62% |  |  |  |
| **Good** | 189 21.09% | 368 24.71% | 219 19.08% |  |  |  |

**Table S3(Continued). Between-class-differences in academic performance and social and demographic background among different parental rearing profiles.**

|  | **Class 1: Positive parental rearing style(n=896)** | **Class 2: Mixture parental rearing style (n =1489)** | **Class 3: Negative rearing style (n =1148)** |  |  |  |
| --- | --- | --- | --- | --- | --- | --- |
|  | **n %** | **n %** | **n %** | **χ^2^** | **p** | **Ф** |
| Excellent | 241 26.90% | 220 14.78% | 176 15.32% |  |  |  |
| Gender |  |  |  | 37.31 | <0.001 | 0.10 |
| Male | 196 21.88% | 296 19.88% | 342 29.79% |  |  |  |
| Female | 700 78.12% | 1193 80.12% | 806 70.21% |  |  |  |
| Poverty |  |  |  | 10.80 | 0.06 | 0.06 |
| Yes | 274 30.58% | 501 33.65% | 318 27.70% |  |  |  |
| No | 622 69.42% | 988 66.35% | 830 72.30% |  |  |  |
|  | **M SD** | **M SD** | **M SD** | **F** | **p** | ***η*^2^** |
| Study hours per week | 27.49 18.38 | 26.41 17.77 | 23.97 22.82 | 2.16 | 0.14 | 34170 |
| Internet hours usage per week | 26.65 16.83 | 26.25 17.54 | 21.41 18.38 | 1.48 | 0.22 | 11321128 |

**Note. χ2 and *F* values were results of the chi-square tests and F-tests.**

**Table S4. characteristic of college students stratified by gender.**

| **Female** | | | | | **Male** | | | | |
| --- | --- | --- | --- | --- | --- | --- | --- | --- | --- |
| Variables | Categories | N or mean | % or SD | χ^2^/p | Variables | Categories | N or  mean | % or SD | χ2/p |
| Rank range | Top 10% | 495 | 18.34 | 711.91^***^ | Rank range | Top 10% | 124 | 14.87 | 127.81^***^ |
|  | 11% – 25% | 756 | 28.02 |  |  | 11% – 25% | 125 | 14.99 |  |
|  | 26% – 50% | 918 | 34.01 |  |  | 26% – 50% | 288 | 34.53 |  |
|  | 51% – 75% | 409 | 15.14 |  |  | 51% – 75% | 183 | 21.94 |  |
|  | Bottom 25% | 121 | 4.49 |  |  | Bottom 25% | 114 | 13.67 |  |
| Subjective family social status | Bottom | 111 | 4.11 | 3422.91^***^ | Subjective family social status | Bottom | 54 | 6.47 | 870.11^***^ |
|  | Low | 469 | 17.38 |  |  | Low | 159 | 19.06 |  |
|  | Middel | 1721 | 63.76 |  |  | Middel | 498 | 59.71 |  |
|  | High | 324 | 12.01 |  |  | High | 77 | 9.23 |  |
|  | Top | 74 | 2.74 |  |  | Top | 46 | 5.53 |  |
| Age (in years) | ≤18 | 51 | 1.89 | 5649.72^***^ | Age (in years) | ≤18 | 25 | 3.00 | 1410.11^***^ |
|  | 18-23 | 2080 | 77.07 |  |  | 18-23 | 597 | 71.58 |  |
|  | 24-29 | 405 | 15.01 |  |  | 24-29 | 108 | 12.95 |  |
|  | 30-35 | 94 | 3.48 |  |  | 30-35 | 62 | 7.43 |  |
|  | ≥36 | 69 | 2.55 |  |  | ≥36 | 42 | 5.04 |  |
| Health status | very poor | 225 | 8.34 | 600.21^***^ | Health status | very poor | 87 | 10.43 | 99.83^***^ |

**Table S4(Continued). characteristic of college students stratified by gender.**

| **Female** | | | | | **Male** | | | | |
| --- | --- | --- | --- | --- | --- | --- | --- | --- | --- |
| **Variables** | **Categories** | **N or mean** | **% or SD** | **χ^2^/p** | **Variables** | **Categories** | **N or**  **mean** | **% or SD** | **χ2/p** |
| Health status | poor | 992 | 36.75 |  |  | poor | 108 | 12.95 |  |
|  | normal | 494 | 18.30 |  |  | normal | 214 | 25.66 |  |
|  | good | 578 | 21.42 |  |  | good | 198 | 23.74 |  |
|  | excellent | 410 | 15.19 |  |  | excellent | 227 | 27.22 |  |
| Poverty | Yes | 908 | 33.64 | 392.49^***^ | Poverty | Yes | 185 | 22.18 | 258.15^***^ |
|  | No | 1791 | 66.36 |  |  | No | 649 | 77.82 |  |
| Major | Arts and Humanities | 939 | 34.79 | 465.07^***^ | Major | Arts and Humanities | 168 | 20.14 | 461.51^***^ |
|  | Medicine | 970 | 35.94 |  |  | Medicine | 472 | 56.59 |  |
|  | Science | 407 | 15.08 |  |  | Science | 85 | 10.19 |  |
|  | Others | 383 | 14.19 |  |  | Others | 109 | 13.08 |  |
| Grade | Freshman | 127 | 4.71 | 1707.31^***^ | Grade | Freshman | 135 | 16.19 | 292.19^***^ |
|  | Sophomore | 1253 | 46.42 |  |  | Sophomore | 360 | 43.17 |  |
|  | Junior | 1168 | 43.28 |  |  | Junior | 291 | 34.89 |  |
|  | Senior | 151 | 5.59 |  |  | Senior | 48 | 5.75 |  |

**Table S4(Continued). characteristic of college students stratified by gender.**

| **Female** | | | | | **Male** | | | | |
| --- | --- | --- | --- | --- | --- | --- | --- | --- | --- |
| **Variables** | **Categories** | **N or mean** | **% or SD** | **χ^2^/p** | **Variables** | **Categories** | **N or**  **mean** | **% or SD** | **χ2/p** |
| Study hours per week | / | 25.69 | 18.35 | 71.84^***^ | Study hours per week | / | 25.11 | 21.49 | 29.07^***^ |
| Internet usage hours per week | / | 26.77 | 18.51 | 73.94^***^ | Internet hours per week | / | 23.42 | 19.13 | 30.32^***^ |

**Note: ***: P<0.001**

**Table S5. Logistic regression examining the association between different profiles of parents rearing styles and mental health issues among male college students (N=834).**

| Mental health problem | No. of exposed cases | Model 1 OR (95 % CI) | Model 2 OR (95 % CI) | Model 3 OR (95 % CI) |
| --- | --- | --- | --- | --- |
| Depression(N=344) | | | | |
| Form 1 | 30 | Ref. | Ref. | Ref. |
| Form 2 | 81 | 2.08(1.12,3.16) | 2.15(1.35,3.49) | 2.13(1.34,3.46) |
| Form 3 | 233 | 11.83(7.63,18.84) | 11.51(7.37,18.49) | 11.40(7.29,18.34) |
| Anxiety(N=398) | | | | |
| Form 1 | 42 | Ref. | Ref. | Ref. |
| Form 2 | 120 | 2.50(1.67,3.81) | 2.41(1.59,3.54) | 2.41(1.60,3.61) |
| Form 3 | 236 | 8.16(5.45,12.43) | 7.49(4.96,11.48) | 7.51(4.97,11.54) |
| Comorbidity(N=313) | | | | |
| Form 1 | 27 | Ref. | Ref. | Ref. |
| Form 2 | 72 | 2.11(1.25,3.21) | 2.01(1.24,3.31) | 2.02(1.24,3.33) |
| Form 3 | 214 | 10.46(6.69,15.19) | 10.01(6.36,14.27) | 10.09(6.39,14.43) |

**Notes: Ref: Reference group.**

**Table S6. Logistic regression examining the association between different profiles of parental rearing styles and mental health issues among female college students (N=2699).**

| Depression(N=748) | No. of exposed cases | Model 1 OR (95 % CI) | Model 2 OR (95 % CI) | Model 3 OR (95 % CI) |
| --- | --- | --- | --- | --- |
| Form 1 | 71 | Ref. | Ref. | Ref. |
| Form 2 | 211 | 1.90(1.44,2.55) | 1.86(1.40,2.32) | 1.85(1.39,2.48) |
| Form 3 | 466 | 12.14(9.21,16.21) | 12.09(9.11,16.22) | 11.87(8.95,15.94) |
| Anxiety(N=948) |  |  |  |  |
| Form 1 | 102 | Ref. | Ref. | Ref. |
| Form 2 | 327 | 2.21(1.73,2.84) | 2.10(1.64,2.70) | 2.12(1.65,2.72) |
| Form 3 | 519 | 10.60(8.24,13.73) | 10.11(7.83,13.16) | 10.09(7.81,13.14) |
| Comorbidity(N=650) |  |  |  |  |
| Form 1 | 51 | Ref. | Ref. | Ref. |
| Form 2 | 169 | 2.10(1.52,2.94) | 2.05(1.48,2.87) | 2.04(1.47,2.86) |
| Form 3 | 430 | 14.55(10.70,20.18) | 14.67(10.71,20.46) | 14.44(10.54,20.16) |

**Note: CI: confidence interval, Ref: Reference group.**

**Table S7. Total, direct and indirect effects of mediators, internet attitude and internet literacy for the association between parental rearing style and mental health problems among male students(N=834).**

|  | Total effect (β*,*95 % CI) | Natural direct effect (β*,*95 % CI) | Natural indirect effect (β*,*95 % CI) | Mediation percentage (%,95 % CI) |
| --- | --- | --- | --- | --- |
| Mediator: Depression(N=344) |  |  |  |  |
| Internet attitude | 0.15(0.12,0.17) | 0.13(0.10,0.15) | 0.01(0.01,0.02) | 6.67(3.16,10.18) |
| Internet literacy | 0.13(0.09,0.16) | 0.12(0.08,0.16) | 0.01(0.01,0.02) | 7.69(3.33,12.06) |
| Mediator: Anxiety(N=398) | |  |  |  |
| Internet attitude | 0.22(0.16,0.27) | 0.20(0.14,0.25) | 0.01(0.01,0.02) | 4.55(2.01,7.08) |
| Internet literacy | 0.20(0.14,0.25) | 0.19(0.13,0.23) | 0.01(0.01,0.02) | 5.00(2.15,7.85) |
| Mediator: Comorbidity(N=313) | | | | |
| Internet attitude | 0.12(0.08,0.17) | 0.12(0.10,0.14) | 0.01(0.01,0.02) | 8.33(3.13,13.54) |
| Internet literacy | 0.12(0.09,0.16) | 0.11(0.08,0.14) | 0.01(0.01,0.02) | 8.33(3.51,13.16) |

**Note: CI: confidence interval, Ref: Reference group.**

**a. All outcomes are binary (eg. depression vs no depression), where positive parental rearing style is the reference group.**

**b. Adjusted for exposure–mediator, mediator–outcome and exposure–outcome confounders (academic performance and social and demographic background factors) as listed in table 4.**

**c. Effect values are standardized regression coefficients (β)**

**d. Mediation percentage = (Natural indirect effect / Natural total effect) × 100%.**

**Table S8. Total, direct and indirect effects of mediators, internet attitude and internet literacy for the association between parental rearing style and mental health problems among female students(N=2699) ^a,b^.**

|  | Total effect (β*,*95 % CI) | Natural direct (β*,*95 % CI) | Natural indirect (β*,*95 % CI) | | Mediation percentage  (%,95 % CI) |
| --- | --- | --- | --- | --- | --- |
| Mediator: Depression (N=748) |  |  | |  |  |
| Internet attitude | 0.08(0.05,0.11) | 0.06(0.03,0.09) | | 0.01(0.01,0.02) | 12.50(4.69,20.31) |
| Internet literacy | 0.08(0.05,0.11) | 0.06(0.03,0.09) | | 0.01(0.01,0.02) | 12.50(4.69,20.31) |
| Mediator: Anxiety (N=948) | | | | | |
| Internet attitude | 0.11(0.09,0.15) | 0.10(0.06,0.15) | | 0.01(0.01,0.02) | 9.09(3.91,14.27) |
| Internet literacy | 0.12(0.08,0.16) | 0.10(0.06,0.14) | | 0.01(0.01,0.02) | 8.33(3.32,13.34) |
| Mediator: Comorbidity (N=650) | | | | | |
| Internet attitude | 0.07(0.04,0.09) | 0.06(0.03,0.08) | | 0.01(0.01,0.02) | 14.29(5.51,23.07) |
| Internet literacy | 0.07(0.04,0.10) | 0.05(0.02,0.09) | | 0.02(0.01,0.02) | 28.58(14.39,42.75) |

**a. All outcomes are binary (eg. depression vs no depression), where positive parental rearing style is the reference group.**

**b. Adjusted for exposure–mediator, mediator–outcome and exposure–outcome confounders (academic performance and social and demographic background factors) as listed in table 4.**

**c. Effect values are standardized regression coefficients (β)**

**d. Mediation percentage = (Natural indirect effect / Natural total effect) × 100%.**

| Mental health problem | No. of exposed cases | Model 1 OR (95 % CI) | Model 2 OR (95 % CI) | Model 3 OR (95 % CI) |
| --- | --- | --- | --- | --- |
| Depression(N=812) | | | | |
| Form 1 | 77 | Ref. | Ref. | Ref. |
| Form 2 | 210 | 1.78(1.35,2.37) | 1.77(1.34,2.36) | 1.79(1.35,2.39) |
| Form 3 | 525 | 12.13(9.27,16.04) | 11.59(8.80,15.42) | 11.67(8.85,15.56) |
| Anxiety(N=995) | | | | |
| Form 1 | 108 | Ref. | Ref. | Ref. |
| Form 2 | 218 | 2.06(1.62,2.64) | 1.96(1.53,2.51) | 2.01(1.57,2.58) |
| Form 3 | 569 | 10.21(7.99,13.15) | 9.15(7.12,11.85) | 9.34(7.26,12.12) |
| Comorbidity(N=708) | | | | |
| Form 1 | 62 | Ref. | Ref. | Ref. |
| Form 2 | 166 | 1.71(1.26,2.13) | 1.69(1.24,2.18) | 1.73(1.26,2.37) |
| Form 3 | 480 | 12.45(9.36,16.64) | 11.92(8.88,15.22) | 12.13(9.02,16.13) |

**Table S9. Logistic regression examining the association between different profiles of parents rearing styles and mental health issues among college students ages 18-24 (N=2677).**

**Note: Ref: Reference group; CI: confidence interval.**

**Table S10. Total, direct and indirect effects of mediators, internet attitude and internet literacy for the association between parental rearing style and mental health problems among college students ages 18-24 (N=2677).**

| Mental health problem | Total effect (β*,*95 % CI) | Natural direct effect (β*,*95 % CI) | Natural indirect effect (β*,*95 % CI) | Mediation percentage (%,95 % CI) |
| --- | --- | --- | --- | --- |
| Mediator:  Depression(N=812)  Internet attitude | 0.11(0.05,0.20) | 0.09(0.06,0.12) | 0.02(0.01,0.03) | 18.18(3.11,33.4) |
| Internet literacy | 0.08(0.04,0.11) | 0.06(0.03,0.09) | 0.02(0.01,0.03) | 25.00(12.11,37.91) |
| Mediator: Anxiety(N=995) |  |  |  |  |
| Internet attitude | 0.12(0.08,0.16) | 0.10(0.07,0.14) | 0.02(0.01,0.04) | 16.67(6.8,26.6) |
| Internet literacy | 0.14(0.10,0.16) | 0.10(0.07,0.13) | 0.02(0.01,0.04) | 14.29(7.1,21.5) |
| Mediator: Comorbidity(N=708) |  |  |  |  |
| Internet attitude | 0.06(0.03,0.09) | 0.05(0.02,0.08) | 0.01(0.01,0.02) | 16.67(5.7, 27.6) |
| Internet literacy | 0.06(0.03,0.09) | 0.05(0.02,0.08) | 0.01(0.01,0.02) | 16.67(5.7, 27.6) |

**Note: CI: confidence interval.**

**a. All outcomes are binary (eg. depression vs no depression), where no depression is the reference group.**

**b. Adjusted for exposure–mediator, mediator–outcome and exposure–outcome confounders (academic performance and social and demographic background factors) as listed in table 5.**

**c. Effect values are standardized regression coefficients (β).**

**d. Mediation percentage = (Natural indirect effect / Natural total effect) × 100%.**

**Table S11. Logistic regression examining the association between different profiles of parents rearing styles and mental health issues among college students who perceived above higher social status(N=521).**

| Mental health problem | No. of exposed cases | Model 1 OR (β*,*95 % CI) | Model 2 OR (β*,*95 % CI) | Model 3 OR (95 % CI) |
| --- | --- | --- | --- | --- |
| Depression(N=123) | | | | |
| Form 1 | 24 | Ref. | Ref. | Ref. |
| Form 2 | 42 | 1.86(1.09,3.05) | 1.95(1.11,3.11) | 1.90(1.08,2.92) |
| Form 3 | 57 | 14.02(7.76,22.17) | 13.40(7.31,21.33) | 13.02(7.08,24.84) |
| Anxiety(N=150) | | | | |
| Form 1 | 32 | Ref. | Ref. | Ref. |
| Form 2 | 54 | 2.34(1.46,3.19) | 2.18(1.53,2.51) | 2.01(1.57,2.58) |
| Form 3 | 64 | 8.73(4.99,12.58) | 9.01(7.12,10.85) | 9.34(7.25,12.12) |
| Comorbidity(N=105) | | | | |
| Form 1 | 17 | Ref. | Ref. | Ref. |
| Form 2 | 38 | 2.42(1.33,4.03) | 2.43(1.34,4.06) | 1.73(1.26,2.37) |
| Form 3 | 50 | 14.78(7.87,28.97) | 14.26(7.49,28.14) | 12.13(9.03,16.53) |

**Note: Ref: Reference group; CI: confidence interval.**

**Table S12. Total, direct and indirect effects of mediators, internet attitude and internet literacy for the association between parental rearing style and mental health problems among college students who perceived above higher social status(N=521).**

| Mental health problem | Total effect (β*,*95 % CI) | Natural direct effect (β*,*95 % CI) | Natural indirect effect (β*,*95 % CI) | Mediation percentage(%,95 % CI) |
| --- | --- | --- | --- | --- |
| Mediator: Depression(N=123) | | | | |
| Internet attitude | 0.14(0.03,0.25) | 0.12(0.02,0.23) | 0.02(0.01.0.03) | 14.29(0.99,27.59) |
| Internet literacy | 0.08(0.03,0.15) | 0.06(0.02,0.10) | 0.02(0.01.0.03) | 25.00(2.46,47.54) |
| Mediator: Anxiety(N=150) | | | | |
| Internet attitude | 0.13(0.03,0.22) | 0.11(0.04,0.20) | 0.02(0.01.0.03) | 15.38(1.77, 29.01) |
| Internet literacy | 0.13(0.05,0.21) | 0.09(0.02,0.18) | 0.03(0.01,0.05) | 23.08(2.15, 44.01) |
| Mediator: Comorbidity(N=105) | | | | |
| Internet attitude | 0.10(0.03,0.15) | 0.09(0.03,0.15) | 0.01(0.01,0.02) | 10.00(2.19,17.81) |
| Internet literacy | 0.16(0.07,0.25) | 0.13(0.04,0.23) | 0.03(0.01,0.05) | 18.75(2.40, 35.10) |

**Note: CI: confidence interval.**

**a. All outcomes are binary (eg. depression vs no depression), where no depression is the reference group.**

**b. Adjusted for exposure–mediator, mediator–outcome and exposure–outcome confounders (academic performance and social and demographic background factors) as listed in table 5.**

**c. Effect values are standardized regression coefficients (β).**

**d.** **Mediation percentage = (Natural indirect effect / Natural total effect) × 100%.**

**APPENDIX A. The dimensions and measurement items of parental rearing style**

**(short form of Egna Minnen av Barndoms Uppfostran (s-EMBU)).**

| **Dimensions** | **Items** |
| --- | --- |
| **Rejection** | 1. It happened that my parents were sour or angry with me without letting me know the cause. |
|  | 2. It happened that my parents gave me more corporal punishment than I deserved. |
|  | 3. My parents criticized me and told me how lazy and useless I was in front of others. |
|  | 4. I was treated as the “black sheep” or “scapegoat” of the family. |
|  | 5. My parents treated me in such a way that I felt ashamed. |
|  | 6.My parents would punish me hard, even for trifles (small offenses). |
| **Emotional**  **warmth** | 7. My parents praised me. |
|  | 8. I think that my parents tried to make my adolescence stimulating, interesting and instructive (for instance by giving me good books, arranging for me to go on camps, taking me to clubs). |
|  | 9. My parents tried to spur me to become the best. |
|  | 10. If things went badly for me, I then felt that my parents tried to comfort and encourage me. |
|  | 11. My parents showed with words and gestures that they liked me. |
|  | 12. I felt that warmth and tenderness existed between me and my parents. |
|  | 13. I felt that my parents were proud when I succeeded in something I had undertaken. |
| **Overprotection** | 14. It happened that I wished my parents would worry less about what I was doing. |
|  | 15. When I came home, I then had to account for what I had been doing to my parents. |
|  | 16. It happened that my parents forbade me to do things other children were allowed to do because they were afraid that something might happen to me. |
|  | 17. I think that my parents’ anxiety that something might happen to me was exaggerated. |
|  | 18. I was allowed to go where I liked without my parents caring too much. |
|  | 19. I felt that my parents interfered with everything I did. |
|  | 20. My parents put decisive limits for what I was and was not allowed to do, to which they then adhered rigorously. |
|  | 21. My parents wanted to decide how I should be dressed or how I should look. |

**APPENDIX B：The dimensions and measurement items of the second digital divide**

| Dimensions | Items |
| --- | --- |
| Internet literacy | 1. I am very proficient in using search engines. |
|  | 2. I am very proficient in using databases to search for literature. |
|  | 3. I can accurately identify false/fraudulent information on the Internet. |
|  | 4. I can install and operate commonly used computer software/mobile apps. |
|  | 5. I have computer programming skills. |
| Internet attitude | 1. I believe the Internet is important to my life. |
|  | 2. I believe the Internet is important to my work. |
|  | 3. I believe the Internet is important to my studies. |

**APPENDIX C：The dimensions and measurement items of anxiety subscale and depression subscale of Depression Anxiety Stress Scale-21.**

| Dimensions | Items |  |
| --- | --- | --- |
| Anxiety | 1.I was aware of the action of my heart in the absence of physical exertion |  |
|  | 2.I experienced breathing difficulty |  |
|  | 3.I experienced trembling |  |
|  | 4.I felt scared without any good reason. |  |
|  | 5.I felt I was close to panic. |  |
|  | 6.I was worried about situations in which I might panic and make a fool of myself. |  |
|  | 7.I was aware of dryness of my mouth. |  |
| **Depression** \| 8. I couldn't seem to experience any positive feeling at all.; \| 9. I found it difficult to work up the initiative to do things.; \| 10. I felt that I had nothing to look forward to.; \| 11. I felt down-hearted and blue.; \| 12. I was unable to become enthusiastic about anything.; \| 13. I felt that I wasn’t worth much as a person.; \| 14. I felt that life was meaningless. | | |
